# Acceptability of quality indicators for the management of endometrial, cervical and ovarian cancer: results of an online survey

**DOI:** 10.1186/s12905-020-00999-3

**Published:** 2020-07-23

**Authors:** Annemie Luyckx, Leen Wyckmans, Anne-Sophie Bonte, Xuan Bich Trinh, Peter A. van Dam

**Affiliations:** 1 Multidisciplinary Oncologic Centre Antwerp (MOCA), Antwerp University Hospital, Wilrijkstraat 10, B2650 Edegem, Belgium; 2 Centre for Oncological Research (CORE), University of Antwerp, B2610 Wilrijk, Belgium

**Keywords:** Quality indicators, Survey, Endometrial cancer, Cervical Cancer, Ovarian Cancer

## Abstract

**Background:**

Measuring quality indicators (QI’s) is a tool to improve the quality of care. The aim of this study was to evaluate the acceptability of 36 QI’s, defined after a literature search for the management of endometrial, cervical and ovarian cancer. Relevant specialists in the field of interest were surveyed.

**Methods:**

To quantify the opinions of these specialists, an online survey was sent out via mailing to members of gynaecological or oncological societies. The relevance of each QI was questioned on a scale from one to five (1 = irrelevant, 2 = less relevant, 3 = no opinion/neutral, 4 = relevant, 5 = very relevant). If a QI received a score of 4 or 5 in 65% or more of the answers, we state that the respondents consider this QI to be sufficiently relevant to use in daily practice.

**Results:**

The survey was visited 238 times and resulted in 53 complete responses (29 Belgian, 24 other European countries). The majority of the specialists were gynaecologists (45%). Five of the 36 QI’s (13,9%) did not reach the cut-off of 65%: referral to a tertiary center, preoperative staging of endometrial cancer by MRI, preoperative staging of cervical cancer by positron-emission tomography, incorporation of intracavitary brachytherapy in the treatment of cervical cancer, reporting ASA and WHO score for each patient. After removing the 5 QI’s that were not considered as relevant by the specialists and 3 additional 3 QI’s that we were considered to be superfluous, we obtained an optimized QI list.

**Conclusion:**

As QI’s gain importance in gynecological oncology, their use can only be of value if they are universally interpreted in the same manner. We propose an optimized list of 28 QI’s for the management of endometrial, cervical and ovarian cancer which responders of our survey found relevant. Further validation is needed to finalize and define a set of QI’s that can be used in future studies, audits and benchmarking.

## Background

During the last decade, there is a growing interest in measuring the quality of care as it has been proven that this has a positive impact on the survival of the patients [1]. In order to improve the standards of care, it is of paramount importance to identify suboptimal elements in the clinical trajectory of a patient. These may be related to factors implicated in the diagnosis, decision making and/or treatment of patients. A tool to expose these deficiencies are quality indicators (QI’s). These are norms, criteria, standards or other direct qualitative and quantitative measures of health care quality that make use of readily available hospital inpatient administrative data [2]. They can highlight potential quality concerns, identify areas that need further study and investigation, track changes over time, and enable physicians to compare their practices with the guidelines and with each other, in order to strive for optimal health care [3, 4]. Recent literature shows that less than 50% of patients suffering from ovarian cancer in Europe are treated according to the guidelines and that there is a correlation between treatment adherent to the guidelines and survival [5]. This is a striking example demonstrating that there is a high need for quality control and benchmarking in our health care systems [4]. Unfortunately, many physicians and hospitals consider measuring QIs an obstacle of their practice and functioning, mainly because of the administrative burden and costs involved [4, 6].

We recently performed a systematic literature search to identify QI’s for the management of patients with endometrial, cervical and ovarian cancer and could select a list of 36 relevant QI’s [7]. We focused on structural, process and outcome measures of health care predominantly for the management of these three cancers. Structural measures refer to health care facility resources, e.g. the number and qualification of staff, case volume of the hospital and specialist, supplies, access to specific technologies etc. [8, 9]. Process measures refer to specific actions implemented to achieve an optimal result, where outcome measures are indicators of the total health of treated patients and the quality of given care [7–9]. Our search showed that important QI’s related to the structural organization of health care were referral to high volume specialists in high volume hospitals, treatment by specialized gynaecological oncologists and discussion of a treatment plan by a multidisciplinary team according to current guidelines. Process measures were adequate pretreatment staging, a patient report of high quality and adherence to treatment guidelines, while outcome indicators were treatment related morbidity and increased survival. In order to be relevant in a clinical setting, the list of 36 selected QI’s list should adequately measure the quality of care, improve the quality of care in daily practice, be cost-effective and contribute to create uniformity.

Optimally, consensus guidelines and QI’s are formulated after a systematic assessment of the literature looking for evidence based healthcare recommendations on how to treat or diagnose a disease with the aim of better patient outcomes [10]. In order to obtain a widely supported workable framework the Delphi method is often used to further refine and define these guidelines and QI’s in a second stage. The Delphi method is a forecasting process framework whereby questionnaires on the subject of interest are send out to a panel of experts asking them to score the relevance of the guidelines. When opinions differ on certain issues, the experts are allowed to adjust their answers anonymously in subsequent rounds based on how they interpret the group response, thereby aiming to work towards a mutual agreement. This was achieved successfully for many tumor types including breast cancer [11, 12]. Although international organizations such as ESMO and ESGO have provided guidelines on the treatment of gynaecological cancers, up to now no agreement has been reached on the proper QI’s which could be used to measure and benchmark the quality of the provided care [7, 13–15]. Therefore, we decided to perform an online survey for specialists involved in the treatment of patients with endometrial, cervical and ovarian cancer to question the acceptability and practicability in daily practice of the QI’s which were distilled out of our literature search [5].

## Methods

### Study population

To evaluate the relevance of the 36 QI’s [10], we designed an online survey (Additional file 1). The questionnaire was sent by electronic mail to all members of the Flemish and Dutch Society of Obstetricians and Gynaecologists, and the Belgian Society of Medical Oncology. A link to the questionnaire with an invitation to participate was also put on the website of the European Society of Gynecological Oncology, and was also available in our paper published in the European Journal of Surgical Oncology [7]. The full survey can be found in the Additional file 1. Participating specialists were informed that they answered the list voluntarily without any rewards and that their anonymized answers would be used for the further course of the study. Since we received less than 50 responses in first 3 months, we sent reminders to all above mentioned specialists.

### Scoring

This survey was created using an online form (my.survio.com) and was accessible from 1 September 2018 to 28 February 2019. The participants were asked to indicate their specialism (gynaecological oncologist, general gynaecologist, medical oncologist, radiotherapist or other) and demographics. The answers of non-European specialists were excluded. The relevance of each QI was questioned on a scale from one to five (1 = irrelevant, 2 = less relevant, 3 = no opinion/neutral, 4 = relevant, 5 = very relevant). Physicians evaluating a QI not belonging to their discipline (eg radiotherapist on surgical issues), had the possibility to not score that QI. In contrast to process measures which are cancer specific, structural and outcome measures applicable for all three cancer types were not questioned separately per cancer type. At the end of the questionnaire, there was room to give remarks and suggestions. For each QI, the ‘Survio’ software recorded all given answers and the frequency of each particular score (1, 2, 3, 4 and 5). Subsequently, the corresponding percentages were calculated. If a QI received a score of 4 or 5 in 65% or more of the answers, we state that the respondents consider this QI to be sufficiently relevant to use in daily practice. The percentage of 65 has been set by mutual agreement (AB, AL, LW, PVD, XBT).

### Statistical analysis

Statistical analysis was performed using the IBM software package, SPSS V.25, and results were graphically displayed with GraphPad prism V.6.0 h.

### Ethical considerations

Ethical approval for the study was obtained by the Ethical Committee of the Antwerp University Hospital (nr. 19/16/215). No human subjects were involved in this study. The physicians responding to the questionnaires agreed that their answers would be used for statistical analysis (see Additional file 1) and publication.

## Results

### Respondents characteristics

Sending out the survey to the specialists resulted in 238 visits and 53 responses. Twenty-nine respondents (54,7%) are working in Belgium and 24 respondents (45.3%) are employed in another European country. The majority of these specialists are gynaecologists (45.3%). The demographic characteristics of the respondents are displayed in Table 1.

**Table 1:** Characteristics of respondents

Characteristic	No. respondents	% total
Sex		
- Male	29	54,7
- Female	24	45,3
Age		
- < 45 years	19	35,8
- > 45 years	34	64,2
Specialism		
- Gynaecological oncologist	15	28,3
- General gynaecologist	24	45,3
- Medical oncologist	8	15,1
- Radiotherapist	5	9,4
- Other	1	1,9
Working country		
- Belgium	29	54,7
- Another country within Europe	24	45,3

### Percentages of given scores

**Fig. 1:**
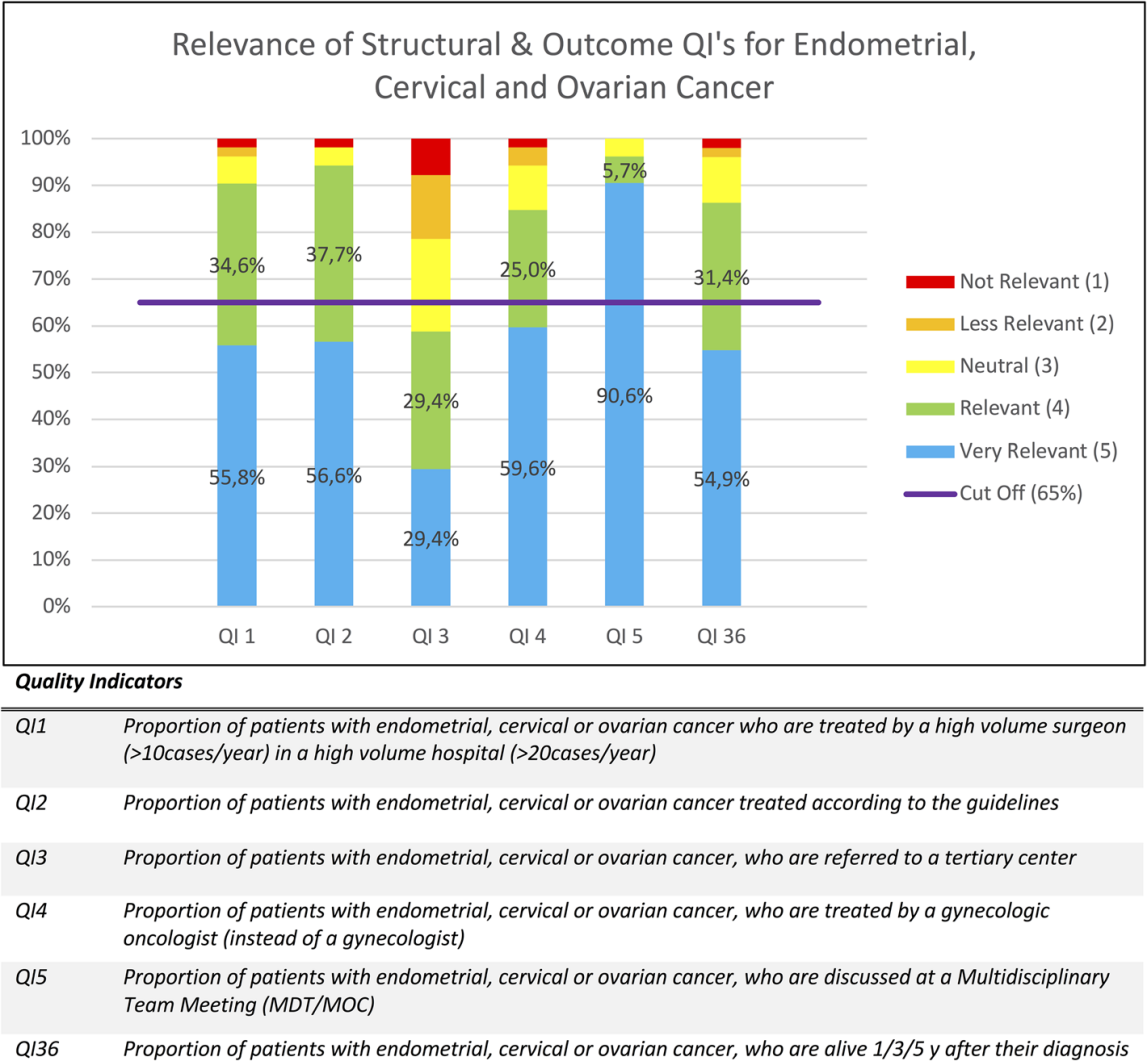
Relevance of structural and outcome quality indicators for endometrial, cervical and ovarian cancer

**Fig. 2:**
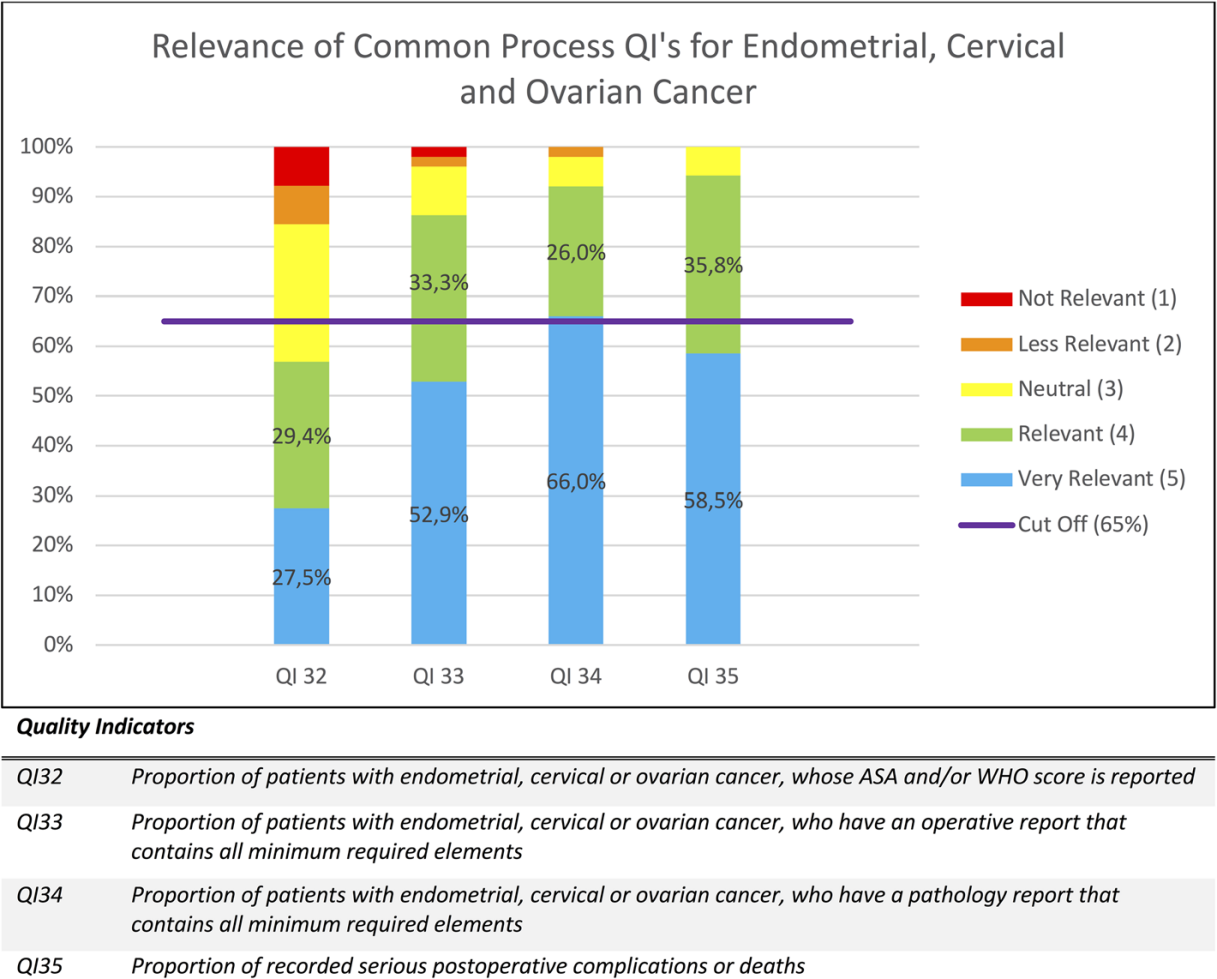
Relevance of common process quality indicators for endometrial, cervical and ovarian cancer

**Fig. 3:**
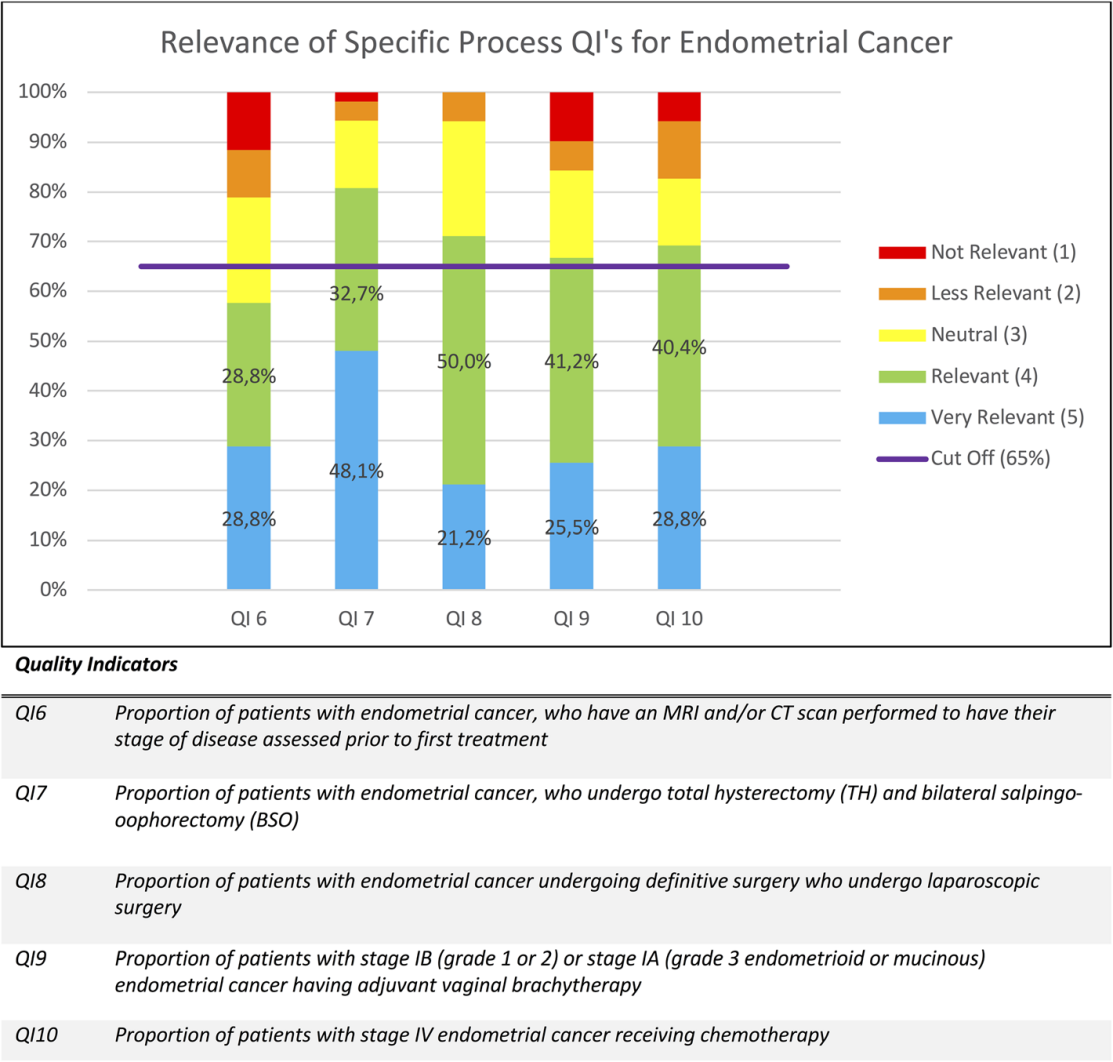
Relevance of specific process quality indicators for endometrial cancer

**Fig. 4:**
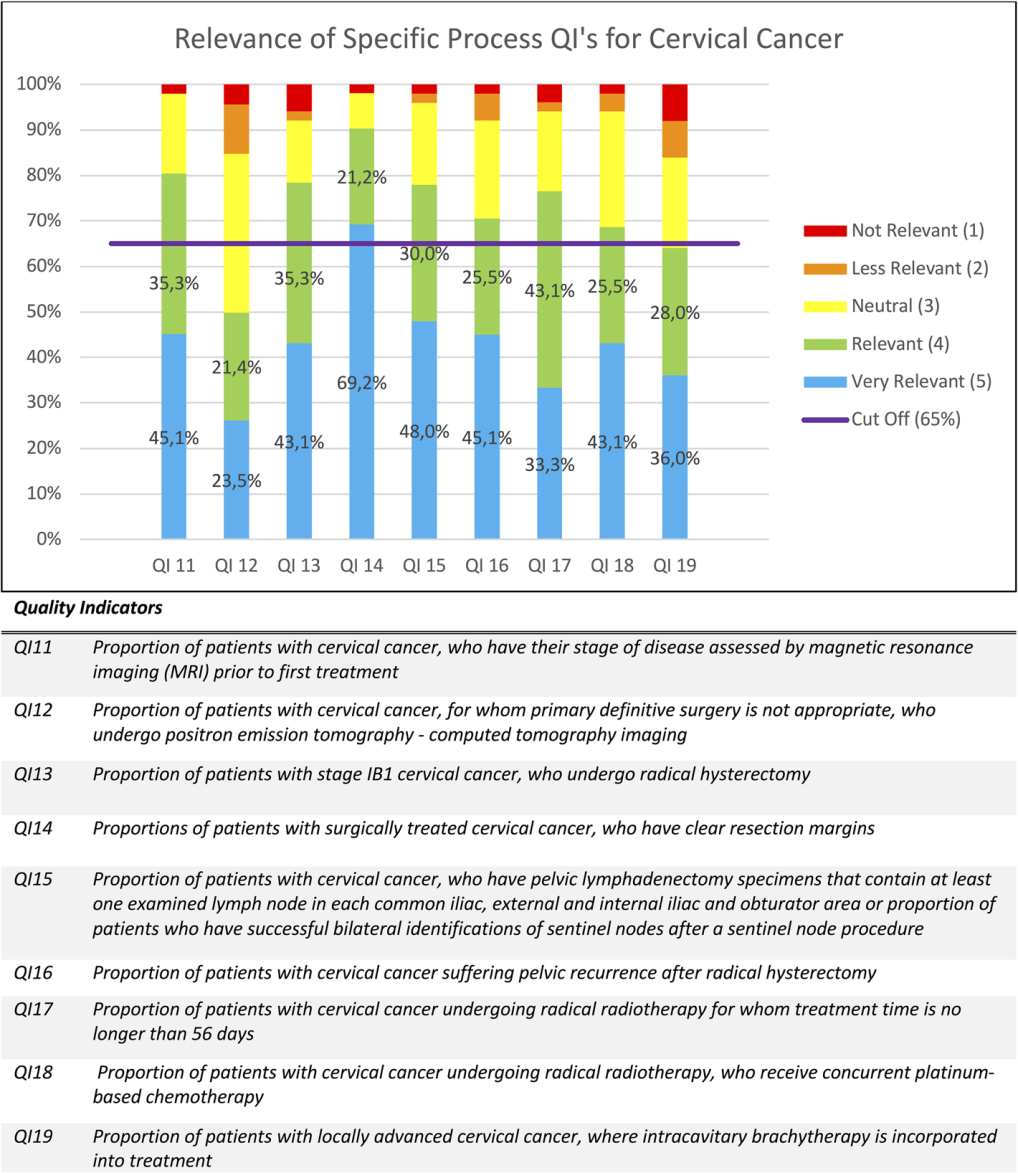
Relevance of specific process quality indicators for cervical cancer

**Fig. 5:**
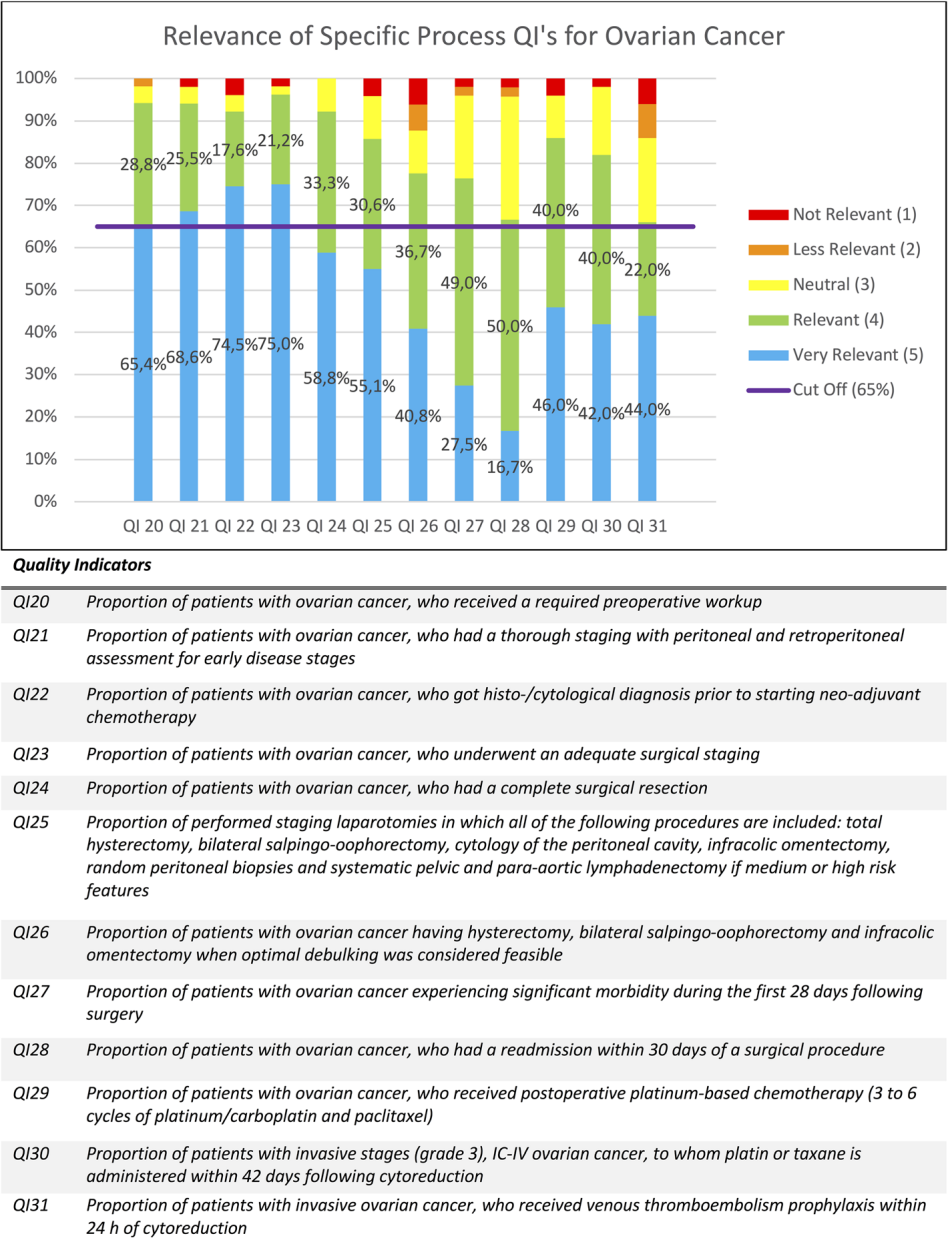
Relevance of specific process quality indicators for ovarian cancer

As mentioned above, the percentages of given scores were calculated for each QI and are displayed in Figs. 1, 2, 3, 4 and 5. Figure 1 contains the results of the structural and outcome QI’s for both endometrium, cervix and ovarian cancer. Figure 2 contains the results of the common process QI’s for the three cancers. The results of the specific process QI’s are displayed in a separate chart per cancer type (Figs. 3, 4 and 5).

### Identification of insufficiently relevant quality indicators

To consider a quality indicator as sufficiently relevant to use in daily practice, we stated that the total percentage of scores 4 and 5 had to be 65% or more. Thirty one of the 36 QI’s (86,1%) reached this cut-off. Seven QI’s were indicated as “very relevant” in 65% or more of the given answers and thus achieved this cut-off with only the percentage of score 5. This concerns QI 5, QI 14, QI 20, QI 21, QI 22, QI 23, QI 34. Five QI’s (13,9%) did not reach the cut-off of 65%: QI 3, QI 6, QI 12, QI 19, QI32. Therefore, we conclude that the specialists considered these QI’s as insufficiently relevant. These five QI’s are discussed in detail below.

## Discussion

Gynaecological cancers are challenging and complex diseases. According to the GLOBOCAN 2018 estimates on the global cancer burden, produced by the International Agency for Research, the incidence and mortality of cervical cancer, cancer of the corpus uteri and ovarian cancer respectively are 569.847, 382.069, 299.414 and 311.365, 89.929, 184.799 [16]. In developed countries, endometrial cancer is the most common of these gynecological cancers and ovarian cancer is the leading cause of death [17, 18]. Although this study mainly focuses on the quality of care to ultimately increase the survival of gynaecological cancer patients, the quality of life should not be omitted. These women have to deal with physical changes, but also with mental and social challenges influencing their overall well-being. The physical changes comprise menopausal symptoms, a changed sexual life, bowel and/or urinary tract problems etc. An important part of the rehabilitation and follow-up of these patients needs to focus on conversations with health professionals around these challenges [19, 20]. In the study of Vitale et al. it is stated that not only young women, but also the elderly are entitled to a standard treatment. Elderly patients are often undertreated because of their many morbidities and low life expectancy, but treatment according to the guidelines can increase their quality of life [21].

Several studies have been performed listing relevant QI’s to measure the quality of cancer care. We were the first to create one list for endometrial, cervical and ovarian cancer [7]. The present survey examined the relevance of the 36 quality indicators which were retained in the above-mentioned literature search. Using an online survey, sent to physicians active in the field, we have shown that 5 of these indicators are not considered to be relevant to measure the quality of care for these three cancers.

“Referral to a tertiary center (QI 3)” is a structural quality indicator that was questioned for all three cancer types by one single statement in our survey. Although the literature supports that there is a survival advantage for patients treated for ovarian and cervical cancers in high volume centers by high volume surgeons, the evidence is much less clear for endometrial cancer [22]. At the end of the questionnaire the specialists had the opportunity to give remarks and suggestions. Some correspondents correctly regretted that they could not score this QI separately for the different tumors types as they considered it relevant for ovarian and cervical cancer. Although surgery is often less complex and the need for adjuvant treatment is often not indicated in endometrial cancer patients with low risk histology, this is not the case in patients with high risk histology. Recent evidence is emerging that in this second group of patients perioperative mortality is higher and survival is reduced in low volume hospitals [22, 23]. It is likely that in a true Delphi procedure this QI would have been debated and rephrased in a second round and accepted for cancer of the ovary, cervix and high risk endometrial cancer.

“Preoperative assessment by MRI and/or computed tomography in patients with endometrial cancer (QI 6)” and “Pretreatment positron emission tomography-computed tomography imaging (PET-CT) should be performed when primary definitive surgery is not appropriate in cervical cancer (QI 12)” are staging process quality indicators. These were based on the opinions of scientific societies but are not feasible in all centers. Although they may have an impact on referral patterns of patients, there is no hard evidence that these issues have a direct impact on patient survival. We therefore dropped them as mandatory QI’s.

According to QI 19, another process indicator, “intracavitary brachytherapy should be incorporated into the treatment of patients with locally advanced cervical cancer”. Our respondents did not accept this QI as mandatory, probably because this technology is not available in all units. It should be mentioned that this QI was the most endorsed QI among specialized radiotherapists, as individual tailored radiotherapy treatment incorporating brachytherapy results in a better outcome of patients [24, 25]. This underscores the need to refer these patients to tertiary centers offering this treatment modality to patients. We therefore would advise to incorporate this QI in future quality control programs.

“Reporting of an ASA and/or WHO score for each patient” (QI 32) is a common process quality indicator for all three cancer types. Although associations between ASA and WHO scores and specific surgical complications or outcomes have been reported in the literature, they can vary over the treatment trajectory of a patient. It was considered complicated by some respondents to register them systematically. To maintain the practicality, this QI was not incorporated in the optimized QI list [26].

The common QI’s must be assessed separately per cancer type. Therefore, they are mentioned separately for each cancer type in the optimized list. Additionally, we have split the outcome QI into a separate QI for 1, 3 and 5-year survival so this can be investigated more accurately.

One of the limitations of the present study is the limited response rate. This is comparable to the proportion of answers recorded after similar electronic surveys on other subjects [27, 28]. Apparently, it is difficult to motivate clinicians to fill in questionnaires voluntarily amid the many other occupations of daily and professional life. However, our respondents seemed to be very motivated. They could make general remarks regarding the entire survey and often did so. We considered every opinion given by a specialist in this field of interest useful as it reflects the vision of clinicians in the real world, which may differ from the view of “experts”. Some of them stated that a few of the QI’s on the list are rather vague, unclear or quite obvious and that some similar QI’s should be combined into one unambiguous QI. We have gone through all QI’s and consequently concluded to remove three additional QI’s from our QI list. The first one is QI2: “Proportion of patients with endometrial, cervical and ovarian cancer treated according to the guidelines”. This QI is indeed superfluous since it is very general, overarching, difficult to measure and it is not specified which guidelines are involved. We want to emphasize that we do think this QI is theoretically very relevant if it is well defined (e.g. according to ESMO guidelines), but we doubt its usefulness in daily practice due to its complexity. In addition, several retained QI’s measure this indirectly. The other two QI’s that we do not preserve in our final list are QI23 and QI25. These process QI’s concern the surgical staging of ovarian tumors. After discussion, we felt that these QI’s are unnecessary because other QI’s cover their content (e.g. QI21 and QI26).

## Conclusions

Our study contributes to the standardizing of healthcare practices, which is essential to deliver consistent and cost-effective healthcare. After removing the 5 QI’s that are not considered as relevant by the specialists and the additional 3 QI’s that we have decided to be superfluous, we obtained an optimized QI list for the management of endometrial, cervical and ovarian cancer (Additional file 2). We hope this paper can initiate a Delphi meeting by experts on this subject finalizing a definite set of QI’s that can be used in a subsequent studies and audits in daily practice.

## Supplementary information

**Additional file 1: Appendix 1.** Survey “Relevance of quality indicators in the management of endometrial, cervical and ovarian cancer”.

**Additional file 2: Appendix 2.** Optimised QI list.

## Data Availability

The datasets used and/or analysed during the current study are available from the corresponding author on reasonable request.

## Abbreviations

QI: Quality Indicator
ESMO: European Society of Medical Oncology
ESGO: European Society of Gynaecological Oncology
GLOBOCAN: Global Cancer Observatory
MRI: Magnetic Resonance Imaging
ASA: American Society of Anesthesiologists
WHO: World Health Organization
