# Supplementary material for: Acceptability of quality indicators for the management of endometrial, cervical and ovarian cancer: results of an online survey
Source: BMC Womens Health. 2020 Jul 23;20:151. doi: 10.1186/s12905-020-00999-3 (PMC7376904; doi:10.1186/s12905-020-00999-3)
Supplement: Supplementary file 1 — Additional file 1: Appendix 1. Survey “Relevance of quality indicators in the management of endometrial, cervical and ovarian cancer”. [file 12905_2020_999_MOESM1_ESM.pdf]

Relevance of quality indicators in the management of endometrium, cervical and ovarium cancer

Bijlage: Enquête

Relevance of quality indicators in the management of endometrium, cervical and ovarium cancer

Geachte Dokter

English version below.

Wij zijn drie masterstudenten geneeskunde aan de Universiteit Antwerpen. In het kader van onze masterproef doen wij onderzoek naar kwaliteitsindicatoren in het beleid van endometrium-, cervix- en ovariumkanker. Tijdens onze literatuurstudie hebben wij een beknopte lijst gemaakt van de meest voorkomende kwaliteitsindicatoren in de huidige literatuur. Deze werden geselecteerd door twee gynaecologische oncologen in het UZA waaronder professor dokter van Dam. Wij zouden deze lijst van kwaliteitsindicatoren graag verder valideren en daarom vragen wij u om volgende enquête in te vullen. Met deze enquête willen wij graag uw opinie over de relevantie van de vooropgestelde kwaliteitsindicatoren bevragen. Onze studie is volledig in het Engels daarom zullen ook alle vragen in het Engels gesteld worden. Per kwaliteitsindicator wordt er gevraagd om de relevantie van deze kwaliteitsindicator te bepalen op een schaal van 1- 5. Indien u geen uitspraak kan doen over de relevantie, gelieve de vraag dan open te laten.

1 = Niet relevant, 2 = Weinig relevant, 3 = Geen mening/neutraal, 4 = Relevant, 5 = Zeer relevant

Door deel te nemen aan deze enquête geeft u toestemming dat we uw antwoorden gebruiken voor onze studie.

Alvast bedankt voor uw medewerking aan onze masterproef!

Met vriendelijke groeten

Anne-Sophie Bonte, Annemie Luyckx, Leen Wyckmans

Dear Doctor

We are three master students in Medicine at the University ofAntwerp. In the context of our master thesis, we investigate the quality indicators in the management of endometrium, cervix and ovarian cancer. During the literature search, we made a concise list of the most common quality indicators in the current literature. These indicators were then selected by two gynecological oncologists at the Antwerp University Hospital, including Professor doctor van Dam. Our intention is to further validate this list of quality indicators, therefore we would like to ask you to fill in the following survey. With this survey we question your opinion on the relevance of the quality indicators. Our study is entirely conducted in English, therefore all questions will be asked in English. For each quality indicator you will be asked to determine the relevance of this quality indicator on a scale of 1-5.

1 = Not relevant, 2 = Less relevant, 3 = No opinion/neutral, 4 = Relevant, 5 = Very relevant

By participating in this survey, you give your permission to use your answers for our study.

Thank you for your cooperation in our master thesis!

Yours sincerely,

Anne-Sophie Bonte, Annemie Luyckx, Leen Wyckmans

1. I work in

- ○ Belgium
- ○ Another country within Europe
- ○ Another country outside Europe

2. My specialism is:

- ○ Gynaecology
- ○ Gynaecologycal oncology
- ○ Medical Oncology
- ○ Radiotherapy
- ○ Other

3. I am part of the age-group:

- ○ < 45 years old
- ○ > 45 years old

4. My sex is:

- ○ Male
- ○ Female

5. QI 1: Proportion of patients with ENDOMETRIAL, CERVICAL OR OVARIAN CANCER who are treated by a high volume surgeon (>10cases/year) in a high volume hospital (>20cases/year)

	1	2	3	4	5	
Not relevant	○	○	○	○	○	Very relevant

6. QI 2: Proportion of patients with ENDOMETRIAL, CERVICAL OR OVARIAN CANCER treated according to the guidelines

	1	2	3	4	5	
Not relevant	○	○	○	○	○	Very relevant

7. QI 3: Proportion of patients with ENDOMETRIAL, CERVICAL OR OVARIAN CANCER, who are referred to a tertiary center

	1	2	3	4	5	
Not relevant	○	○	○	○	○	Very relevant

8. QI 4: Proportion of patients with ENDOMETRIAL, CERVICAL OR OVARIAN CANCER, who are treated by a gynecologic oncologist (instead of a gynecologist)

	1	2	3	4	5	
Not relevant	○	○	○	○	○	Very relevant

9. QI 5: Proportion of patients with ENDOMETRIAL, CERVICAL OR OVARIAN CANCER, who are discussed at a Multidisciplinary Team Meeting (MDT/MOC)

	1	2	3	4	5	
Not relevant	○	○	○	○	○	Very relevant

10. QI 6: Proportion of patients with ENDOMETRIAL CANCER, who have an MRI and/or CT scan performed to have their stage of disease assessed prior to first treatment

	1	2	3	4	5	
Not relevant	○	○	○	○	○	Very relevant

11. QI 7: Proportion of patients with ENDOMETRIAL CANCER, who undergo total hysterectomy (TH) and bilateral salpingo-oophorectomy (BSO)

	1	2	3	4	5	
Not relevant	○	○	○	○	○	Very relevant

12. QI 8: Proportion of patients with ENDOMETRIAL CANCER undergoing definitive surgery who undergo laparoscopic surgery

	1	2	3	4	5	
Not relevant	○	○	○	○	○	Very relevant

13. QI 9: Proportion of patients with STAGE IB (grade 1 or 2) OR STAGE IA (grade 3 endometrioid or mucinous) ENDOMETRIAL CANCER having adjuvant vaginal brachytherapy

	1	2	3	4	5	
Not relevant	○	○	○	○	○	Very relevant

14. QI 10: Proportion of patients with STAGE IV ENDOMETRIAL CANER receiving chemotherapy

	1	2	3	4	5	
Not relevant	○	○	○	○	○	Very relevant

15. QI 11: Proportion of patients with CERVICAL CANCER, who have their stage of disease assessed by magnetic resonance imaging (MRI) prior to first treatment

	1	2	3	4	5	
Not relevant	◯	◯	◯	◯	◯	Very relevant

16. QI 12: Proportion of patients with CERVICAL CANCER, for whom primary definitive surgery is not appropriate, who undergo positron emission tomography - computed tomography imaging

	1	2	3	4	5	
Not relevant	◯	◯	◯	◯	◯	Very relevant

17. QI 13: Proportion of patients with STAGE IB1 CERVICAL CANCER, who undergo radical hysterectomy

	1	2	3	4	5	
Not relevant	◯	◯	◯	◯	◯	Very relevant

18. QI 14: Proportions of patients with surgically treated CERVICAL CANCER, who have clear resection margins

	1	2	3	4	5	
Not relevant	◯	◯	◯	◯	◯	Very relevant

19. QI 15: Proportion of patients with CERVICAL CANCER, who have pelvic lymphadenectomy specimens that contain at least one examined lymph node in each common iliac, external and internal iliac and obturator area or proportion of patients who have successful bilateral identifications of sentinel nodes after a sentinel node procedure

	1	2	3	4	5	
Not relevant	◯	◯	◯	◯	◯	Very relevant

20. QI 16: Proportion of patients with CERVICAL CANCER suffering pelvic recurrence after radical hysterectomy

	1	2	3	4	5	
Not relevant	◯	◯	◯	◯	◯	Very relevant

21. QI 17: Proportion of patients with CERVICAL CANCER undergoing radical radiotherapy for whom treatment time is no longer than 56 days

	1	2	3	4	5	
Not relevant	○	○	○	○	○	Very relevant

22. QI 18: Proportion of patients with CERVICAL CANCER undergoing radical radiotherapy, who receive concurrent platinum-based chemotherapy

	1	2	3	4	5	
Not relevant	○	○	○	○	○	Very relevant

23. QI 19: Proportion of patients with LOCALLY ADVANCED CERVICAL CANCER, where Intracavitary brachytherapy is incorporated into treatment

	1	2	3	4	5	
Not relevant	○	○	○	○	○	Very relevant

24. QI 20: Proportion of patients with OVARIAN CANCER, who received a required preoperative workup

	1	2	3	4	5	
Not relevant	○	○	○	○	○	Very relevant

25. QI 21: Proportion of patients with OVARIAN CANCER, who had a thorough staging with peritoneal and retroperitoneal assessment for early disease stages

	1	2	3	4	5	
Not relevant	○	○	○	○	○	Very relevant

26. QI 22: Proportion of patients with OVARIAN CANCER, who got histo/cytological diagnosis prior to starting neo-adjuvant chemotherapy

	1	2	3	4	5	
Not relevant	○	○	○	○	○	Very relevant

27. QI 23: Proportion of patients with OVARIAN CANCER, who underwent an adequate surgical staging

	1	2	3	4	5	
Not relevant	○	○	○	○	○	Very relevant

28. QI 24: Proportion of patients with OVARIAN CANCER, who had a complete surgical resection

	1	2	3	4	5	
Not relevant	○	○	○	○	○	Very relevant

29. QI 25: Proportion of performed staging laparotomies in which all of the following procedures are included: total hysterectomy, bilateral salpingo-oophorectomy, cytology of the peritoneal cavity, infracolic omentectomy, random peritoneal biopsies and systematic pelvic and para-aortic lymphadenectomy if medium or high risk features

	1	2	3	4	5	
Not relevant	○	○	○	○	○	Very relevant

30. QI 26: Proportion of patients with OVARIAN CANCER having hysterectomy, bilateral salpingo-oophorectomy and infracolic omentectomy when optimal debulking was considered feasible

	1	2	3	4	5	
Not relevant	○	○	○	○	○	Very relevant

31. QI 27: Proportion of patients with OVARIAN CANCER experiencing significant morbidity during the first 28 days following surgery

	1	2	3	4	5	
Not relevant	○	○	○	○	○	Very relevant

32. QI 28: Proportion of patients with OVARIAN CANCER, who had a readmission within 30 days of a surgical procedure

	1	2	3	4	5	
Not relevant	○	○	○	○	○	Very relevant

33. QI 29: Proportion of patients with OVARIAN CANCER, who received postoperative platinum-based chemotherapy (3 to 6 cycles of platinum/carboplatin and paclitaxel)

	1	2	3	4	5	
Not relevant	○	○	○	○	○	Very relevant

34. QI 30: Proportion of patients with INVASIVE STAGES (grade 3), IC-IV OVARIAN CANCER, to whom platin or taxane is administered within 42 days following cytoreduction

	1	2	3	4	5	
Not relevant	○	○	○	○	○	Very relevant

35. QI 31: Proportion of patients with invasive OVARIAN CANCER, who received venous thromboembolism prophylaxis within 24 h of cytoreduction

	1	2	3	4	5	
Not relevant	○	○	○	○	○	Very relevant

36. QI 32: Proportion of patients with ENDOMETRIAL, CERVICAL OR OVARIAN CANCER, whose ASA and/or WHO score is reported

	1	2	3	4	5	
Not relevant	○	○	○	○	○	Very relevant

37. QI 33: Proportion of patients with ENDOMETRIAL, CERVICAL OR OVARIAN CANCER, who have an operative report that contains all minimum required elements

	1	2	3	4	5	
Not relevant	○	○	○	○	○	Very relevant

38. QI 34: Proportion of patients with ENDOMETRIAL, CERVICAL OR OVARIAN CANCER, who have a pathology report that contains all minimum required elements

	1	2	3	4	5	
Not relevant	○	○	○	○	○	Very relevant

39. QI 35: Proportion of recorded serious postoperative complications or deaths

	1	2	3	4	5	
Not relevant	○	○	○	○	○	Very relevant

40. QI 36: Proportion of patients with ENDOMETRIAL, CERVICAL OR OVARIAN CANCER, who are alive 1/3/5 y after their diagnosis

	1	2	3	4	5	
Not relevant	○	○	○	○	○	Very relevant

41. Do you have any remarks regarding this survey or the quality indicators presented above?
