# Supplementary material for: Acceptability of quality indicators for the management of endometrial, cervical and ovarian cancer: results of an online survey
Source: BMC Womens Health. 2020 Jul 23;20:151. doi: 10.1186/s12905-020-00999-3 (PMC7376904; doi:10.1186/s12905-020-00999-3)
Supplement: Supplementary file 2 — Additional file 2: Appendix 2. Optimised QI list. [file 12905_2020_999_MOESM2_ESM.pdf]

Appendix 2: Optimised QI list

	ENDOMETRIAL CANCER - Quality indicators
**Structural QI's**	
	**QI 1:** Proportion of patients who are treated by a high volume surgeon (>10cases/year) in a high volume hospital (>20cases/year)
	**QI 2:** Proportion of patients who are treated by a gynecologic oncologist (instead of a gynecologist)
	**QI 3:** Proportion of patients who are discussed at a Multidisciplinary Team Meeting (MDT/MOC)
**Process QI's**	
Peri-operative	**QI 4:** Proportion of patients who undergo total hysterectomy (TH) and bilateral salpingo-oophorectomy (BSO)
	**QI 5:** Proportion of patients undergoing definitive surgery who undergo laparoscopic surgery
Non-operative	**QI 6:** Proportion of patients with stage IB (grade 1 or 2) or stage IA (grade 3 endometrioid or mucinous) endometrial cancer having adjuvant vaginal brachytherapy
	**QI 7:** Proportion of patients with stage IV endometrial cancer receiving chemotherapy
Patient report	**QI 8:** Proportion of patients who have an operative report that contains all minimum required elements
	**QI 9:** Proportion of patients who have a pathology report that contains all minimum required elements
	**QI 10**: Proportion of recorded serious postoperative complications or deaths
**Outcome QI's**	
Survival	**QI 11:** Proportion of patients who are alive 1 year after their diagnosis
	**QI 12:** Proportion of patients who are alive 3 years after their diagnosis
	**QI 13:** Proportion of patients who are alive 5 years after their diagnosis

	**CERVICAL CANCER - Quality indicators**
**Structural QI's**	
	**QI 1:** Proportion of patients who are treated by a high volume surgeon (>10cases/year) in a high volume hospital (>20cases/year)
	**QI 2:** Proportion of patients who are treated by a gynecologic oncologist (instead of a gynecologist)
	**QI 3:** Proportion of patients who are discussed at a Multidisciplinary Team Meeting (MDT/MOC)
**Process QI's**	
Pre-operative	**QI 14:** Proportion of patients who have their stage of disease assessed by magnetic resonance imaging (MRI) prior to first treatment
Peri-operative	**QI 15:** Proportion of patients with stage IB1 cervical cancer, who undergo radical hysterectomy
	**QI 16:** Proportions of patients with surgically treated cervical cancer who have clear resection margins
	**QI 17:** Proportion of patients who have pelvic lymphadenectomy specimens that contain at least one examined lymph node in each common iliac, external and internal iliac and obturator area or proportion of patients who have successful bilateral identifications of sentinel nodes after a sentinel node procedure
	**QI 18:** Proportion of patients suffering pelvic recurrence after radical hysterectomy for cervical cancer
Non-operative	**QI 19:** Proportion of patients undergoing radical radiotherapy for whom treatment time is no longer than 56 days
	**QI 20:** Proportion of patients undergoing radical radiotherapy, who receive concurrent platinum-based chemotherapy
Patient report	**QI 8**: Proportion of patients who have an operative report that contains all minimum required elements
	**QI 9:** Proportion of patients who have a pathology report that contains all minimum required elements
	**QI 10:** Proportion of recorded serious postoperative complications or deaths
**Outcome QI's**	
Survival	**QI 11:** Proportion of patients who are alive 1 year after their diagnosis
	**QI 12:** Proportion of patients who are alive 3 years after their diagnosis
	**QI 13:** Proportion of patients who are alive 5 years after their diagnosis

	**OVARIAN CANCER - Quality indicators**
**Structural QI's**	
	**QI 1:** Proportion of patients who are treated by a high volume surgeon (>10cases/year) in a high volume hospital (>20cases/year)
	**QI 2:** Proportion of patients who are treated by a gynecologic oncologist (instead of a gynecologist)
	**QI 3:** Proportion of patients who are discussed at a Multidisciplinary Team Meeting (MDT/MOC)
**Process QI's**	
Pre-operative	**QI 21:** Proportion of patients who received a required preoperative workup
	**QI 22:** Proportion of patients who had a thorough staging with peritoneal and retroperitoneal assessment for early disease stages
	**QI 23:** Proportion of patients who got histo-/cytological diagnosis prior to starting neo-adjuvant chemotherapy
Peri-operative	**QI 24:** Proportion of patients who had a complete surgical resection
	**QI 25:** Proportion of patients having hysterectomy, bilateral salpingo-oophorectomy and infracolic omentectomy when optimal debulking was considered feasible
Post-operative	**QI 26:** Proportion of patients with ovarian cancer experiencing significant morbidity during the first 28 days following surgery.
	**QI 27:** Proportion of patients who had a readmission within 30 days of a surgical procedure
Non-operative	**QI 28:** Proportion of patients with ovarian cancer who received postoperative platinum-based chemotherapy (3 to 6 cycles of platinum/carboplatin and paclitaxel)
	**QI 29:** Proportion of patients with invasive stages I (grade 3), IC-IV ovarian, fallopian tube, or peritoneal cancer to whom platin or taxane is administered within 42 days following cytoreduction
	**QI 30:** Proportion of patients with invasive ovarian, fallopian tube or peritoneal cancer who received venous thromboembolism prophylaxis within 24 h of cytoreduction
Patient report	**QI 8:** Proportion of patients who have an operative report that contains all minimum required elements
	**QI 9:** Proportion of patients who have a pathology report that contains all minimum required elements
	**QI 10:** Proportion of recorded serious postoperative complications or deaths

**Outcome QI's**	
Survival	**QI 11:** Proportion of patients who are alive 1 year after their diagnosis
	**QI 12:** Proportion of patients who are alive 3 years after their diagnosis
	**QI 13:** Proportion of patients who are alive 5 years after their diagnosis
